# Factor structure of the SDQ and longitudinal associations from pre-school to pre-teen in New Zealand

**DOI:** 10.1371/journal.pone.0247932

**Published:** 2021-03-11

**Authors:** John M. D. Thompson, Rebecca F. Slykerman, Clare R. Wall, Rinki Murphy, Edwin A. Mitchell, Karen E. Waldie

**Affiliations:** 1 Department of Paediatrics: Child and Youth Health, Faculty of Medical and Health Sciences, University of Auckland, Auckland, New Zealand; 2 Department of Obstetrics and Gynaecology, Faculty of Medical and Health Sciences, University of Auckland, Auckland, New Zealand; 3 Department of Psychological Medicine, Faculty of Medical and Health Sciences, University of Auckland, Auckland, New Zealand; 4 Discipline of Nutrition, Faculty of Medical and Health Sciences, University of Auckland, Auckland, New Zealand; 5 Department of Medicine, Faculty of Medical and Health Sciences, University of Auckland, Auckland, New Zealand; 6 School of Psychology, Faculty of Science, University of Auckland, Auckland, New Zealand; Chiba Daigaku, JAPAN

## Abstract

**Objective:**

The objective of this study was to assess the validity of the Strengths and Difficulties Questionnaire in a cohort of New Zealand children followed from birth to the age of eleven. The study also aimed to assess the stability of the child data in relation to behavioural outcomes during this period.

**Methods:**

Children in the Auckland Birthweight Collaborative Study were assessed at approximately 3½, 7 and 11 years of age. At all time-points parents completed the parent version of the Strengths and Difficulties Questionnaire, and the children themselves completed the self-report version at 11 years of age. The validity and internal consistency were assessed using exploratory factor analysis, Cronbach’s alpha, and McDonald’s Omega. Cross tabulations and Chi-square statistics were used to determine whether Total Difficulty scores, as per accepted cut-offs, remained stable over time (between normal and abnormal/borderline categories).

**Results:**

The factor structure remained relatively consistent across all three time-points, though several questions did not load as per the originally published factor analysis at the earliest age. The internal consistency of the Strengths and Difficulties Questionnaire was good at all time-points and for parent- and child-completed versions. There was low agreement in the total scores between time points.

**Conclusions:**

The factor analysis shows that the Strengths and Difficulties Questionnaire has a similar factor structure, particularly in older ages, to that previously published and shows good internal consistency. At the pre-school follow up, a larger than expected proportion of children were identified with high scores, particularly in the conduct sub-scale. Children’s behaviour changes over time, with only poor to moderate agreement between those identified as abnormal or borderline over the longitudinal follow up.

## Introduction

In 1997 Goodman published a behavioural screening questionnaire which was designed to collect measures of contemporary issues in child development and was shown to be highly correlated with the Rutter questionnaires [1]. The newly developed Strengths and Difficulties Questionnaire (SDQ) measures five constructs (hyperactivity, emotion, conduct, peer problems and pro-social) and included a total score, which sums the first four of these sub-scales. Since this time the SDQ has become widely used, both in clinical practice and research, and has been translated and validated in numerous languages around the world e.g Nordic languages [2, 3], German [4], Dutch [5] and Chinese, though the structure of the scales has been questioned in this population [6].

In a large study assessing the validity of the teacher, parent and self-completed questionnaires, Goodman carried out a factor analysis that shows the relative weighting of each question to the various sub-scores, and the reliability coefficients. Weightings were generally found to be of a similar size for each component of the sub-scores, generally between 0.6 and 0.7, with a few extra factors loading onto scores above 0.3 but below the loadings of the anticipated contributory questions. Reliability scores for the sample were good with the total at 0.8 and the sub scores between 0.57 for peer problems and 0.77 for hyperactivity [7]. Similar reliability has also been shown in other populations [8, 9] and ages [10, 11]. A review with 48 studies confirmed that the SDQ is a psychometrically sound measure of child mental health and behavioural problems [12] and can be used to predict standardised academic performance [11] and psychopathology [13]. Data from a large sample of 3–5 year old German preschool children also confirmed the 5-factor solution as the best fit of the data, and confirmed the usefulness of the screening tool for young children [14].

In a recent study by Costa and colleagues (2020), the psychometric properties of the parent version of the SDQ in a Portuguese community sample were examined [15]. Children’s ages ranged from 6 to 18 years (mean age =  11, s.d. =  3). They found support for a five-factor model, but failed to find evidence of invariance between genders [15]. Alternatively, Ortuño-Sierra et al. (2018) found both a five-factor model fit and invariance of the SDQ scores across both gender and educational level in a parent sample from the Spanish National Survey [16]. The mean age of children was 9.13 (*SD* = 3.21) with ages ranging from 4 to 14. With regard to the child self-report SDQ, Ortuño-Sierra et al. (2018) found that a five-factor model best suited the data [17]. They also found measurement invariance across both gender and in Spanish adolescents (mean age = 15.15, s.d. = 1.99) [17].

Not all studies have found support for the five-factor model, Goodman himself [13] proposed a three-factor model as an alternate to the five subscales: 1) Internalizing symptoms (from the Emotional and Peer Problems subscales); 2) Externalizing symptoms (from the Conduct Problems and Hyperactivity subscales); and 3) the Prosocial subscale [13]. A similar structure has also been proposed by Ruchkin et al [18]. Other researchers have found support for a four factor model [9] and even a bifactor model [19]. It is clear that, despite the growing body of research into the psychometric properties of the SDQ, further research is needed, particularly with the child version across multiple time points [20] and there are few studies of measurement invariance in the self-reported version of the SDQ [18].

These validation studies and indeed most of the research using the SDQ as a behavioural measure have taken place in cross-sectional studies of children ranging in age from 3 to 16 years. One study from Norway has looked at changes in SDQ scores in the youth part of the Oslo Health Study over a 3-year period from 15 to 18 years of age [21]. That study reported that the internal consistency of the study at baseline and follow-up to be 0.73 and 0.77 for the total difficulties scores, but pointed out that the SDQ has not been validated in 18–19 year olds. They found increases in pro-social behaviour scores for Norwegian and ethnic minorities for both boys and girls and decreases in conduct problems except for ethnic minority girls. They also saw increases in hyperactivity-inattentive scores for all groups except Norwegian boys, and decreased peer problems amongst Norwegian children [21].

Researchers from another longitudinal study (the German BELLA mental health study) administered the SDQ data over a 6-year period, in children and adolescents ranging in age from 9 to 15 years. They found that, overall, 20% of the sample were classified with Total Difficulties as either “Borderline” or “Abnormal.” Low SES was a risk factor for increased scores. There were sex effects, with females more likely than males to have emotional problems, and males more likely to have high scores on the hyperactivity subscale. Over time, 80% of individuals classified as “normal” at baseline remained normal. In general, emotional and behavioral problems persisted over the 6-year period, with those rated as “borderline” at baseline most likely to change diagnostic category. The hyperactivity subscale was most likely to normalize over time, which the authors argue might be due to either treatment effects or spontaneous remission of symptoms due to neuronal development [22].

The Auckland Birthweight Collaborative (ABC) Study [23] has previously shown that the conduct subscale scores in New Zealand 3½ year old children appears to be high, and hence a large proportion of children are defined as being abnormal [24]. Further longitudinal information on the course of emotional and behavioural problems in childhood is important for both clinical practice and to our understanding of human development.

The aims of this analysis was to 1) Explore the factor structure of the SDQ in a cohort of children at various ages, 2) to determine the, internal consistency of the SDQ longitudinally in this cohort of children, and 3) to assess the stability of the identification of those with abnormal/borderline scores using SDQ in the same cohort of children at 3½, 7, and 11 years of age.

## Method

### Participants

The Auckland Birthweight Collaborative Study is a longitudinal study based on a case-control study of small for gestational age infants. The study has been described in detail elsewhere [23], however in brief all small for gestational age infants born and resident between 16 October 1995 and 30 November 1997 in the Auckland district health board and between 16 October 1995 and 26 August 1996 in Waitemata district health boards were eligible to take part. A random sample of appropriate for gestational age infants in both district health boards over the same time-periods formed the control group.

The study collected information at birth from 1714 eligible mothers and their babies, of these 871 mothers were identified from the obstetric records as identifying as European. Due to poor response at early follow-ups (1 and 3½ years) from non-European groups, the study has been restricted to the European group since this time. This analysis is also restricted to European to allow direct comparison across the time-periods. Follow up at 3½ years was carried out on 550 children, at 7 years 591 children and most recently 620 children at 11 years of age.

Each phase of the study received ethical approval from the Auckland Regional Ethics Committee. Initial recruitment at birth (95/084), 3.5-year-old follow-up (99/097), 7 year old follow-up (AKX/02/00/319) and 11 year old follow-up (NTY/11/11/098). The parents of the children gave signed consent for the study, with the children themselves giving assent at 11 years of age.

### Instruments

As part of these follow-ups the parents of the children completed the parent version of the Strengths and Difficulties Questionnaire (SDQ) and in the most recent follow up the children completed the self-report version of the form [7]. No teacher versions of the forms have been obtained during these follow-ups.

In brief, the SDQ is a 25-question tool to assess common difficulties in children. It is scored on a 3-point (0, 1, 2) Likert scale relating to never, sometimes and usually. Several items are reversed which is accounted for in the scoring algorithm. The questionnaire comprises of five subscales (Emotion, Conduct, Hyperactivity, Peer Problems, and Prosocial) each with five items. The first four sub-scales are summed to give a total score (range 0 to 40), the fifth sub-scale (Prosocial) is a positively orientated scale and assessed separately. Each subscale and the total scores have standardised cut-offs derived from population norms to determine abnormal (10%), borderline (10%) and normal (80%) [7]. For the purposes of analysis, the abnormal and borderline groups are often combined (particularly with smaller samples sizes), and this approach was used in this analysis.

### Statistical methods

The questionnaires at each age were assessed using exploratory factor analysis using 5 factors (as this is the pre-defined number of sub-scales of the SDQ). Five factors were also indicated as the suitable number of factors based on the scree plots). We used a varimax rotation to assess the validity of the factor structure of the SDQ questionnaire in the New Zealand population, at each age, and to determine whether there is any difference over time in the weightings of each question.

The internal consistency of the total score and each of the subscales has been assessed using Cronbach’s alpha and to assess any bias that may exist in this measure we also assessed McDonalds Omega.

The correlation of the parental questionnaires over time and between the parent and children’s questionnaires at 11 were assessed using Pearson’s correlation co-efficient for the continuous scores and by kappa statistics using the abnormal/normal categorisation. All analyses were carried out using SAS v9.4 (SAS Institute, Cary, NC), McDonalds Omega was calculated using a SAS Macro as described by Hayes and Coutts [25].

## Results

### Factor structure of SDQ parental report at 3½ years of age

Table 1 shows the factor analysis for the parent questionnaire when the child was between 3½ and 4 and for when the child was between 7 and 8 years of age. At the 3½ year old follow up, 22 of the items loaded onto the predicted factors with loadings of 0.4 or more. Those that didn’t were the “obeys” item in the conduct sub-scale (loading = 0.37), “reflect” in the hyperactivity sub-scale (loading = 0.37), and”bullied” on the peer problems sub-scale (loading = 0.32), the latter 2 did however have higher loadings on the hyperactivity factor than any other remaining item. The conduct sub-scale had three other items with similar loadings. In addition, the pro-social sub-scale had a high negative loading with “reflect” but this loading was smaller in magnitude than the expected items.

**Table 1:** Factor analysis of parent SDQ at 3½ and 7 years of age.

		Parent SDQ at 3½ years					Parent SDQ at 7 years				
		Factor 1	Factor 2	Factor 3	Factor 4	Factor 5	Factor 1	Factor 2	Factor 3	Factor 4	Factor 5
Conduct	Tantrums	**0.59**					**0.46**				-0.34
	Obeys	0.37				-0.39	0.22		0.31		**-0.51**
	Fights	**0.69**					**0.66**				
	Lies	**0.64**					**0.66**				
	Steals	**0.62**					**0.66**				
Emotion	Somatic		**0.48**					**0.47**			
	Worries		**0.68**					**0.77**			
	Unhappy	0.37	**0.50**					**0.53**			
	Clingy		**0.59**					**0.69**			
	Afraid		**0.69**					**0.74**			
Hyperactivity	Restless	0.30		**0.74**					**0.82**		
	Fidgety	0.38		**0.66**					**0.78**		
	Distractible			**0.73**					**0.80**		
	Reflective			0.37		**-0.47**			**0.48**		-0.37
	Attends			**0.68**					**0.67**		
Peer Problems	Bullied		0.32		0.32			0.31		**0.42**	
	Popular				**0.52**	-0.32				**0.59**	-0.31
	Good Friend				**0.67**					**0.61**	
	Loner				**0.58**					**0.63**	
	Relationships				**0.64**					**0.64**	
Prosocial	Considerate					**0.62**					**0.71**
	Shares					**0.50**					**0.55**
	Caring					**0.66**					**0.70**
	Kind					**0.56**					**0.51**
	Helps out					**0.66**					**0.71**

### Factor structure of SDQ parental report at 7 years of age

The results at 7 were more in line with loadings previously reported and are generally larger in magnitude than seen in the 3½ year old data. Only one item did not load as for the initial factor structure and that was again the “obeys” item on the conduct sub-scale, though as with the 3½ year old data the loading was higher than for any other remaining item. The pro-social sub-scale again had a high negative loading for an additional item (“obeys”) whose loading was similar to the smallest loading of the expected items.

### Factor structure of SDQ parental report and child self-report at 11 years of age

Table 2 shows the factor analyses for the parent and children’s questionnaires at 11 years of age. For the parent questionnaire, all 25 items loaded as initially published and only the conduct subscale had an additional item (“unhappy”) with a loading similar to the lowest weighting of the expected items. For the children’s questionnaire two items did not load as expected, “obeys” on the conduct (loading = 0.25) and three other items had higher loadings than this. The “bullied” item of the peer problems subscale (loading = 0.37) had a slightly lower than the “lies” item.

**Table 2:** Factor analysis of parent and child SDQ at 11 years of age.

		Parent SDQ at 11 years					Child SDQ at 11 years				
		Factor 1	Factor 2	Factor 3	Factor 4	Factor 5	Factor 1	Factor 2	Factor 3	Factor 4	Factor 5
Conduct	Tantrums	**0.57**					**0.53**				
	Obeys	**0.41**		0.30			0.25		**0.48**		
	Fights	**0.62**					**0.62**				
	Lies	**0.67**					**0.47**		0.31	0.43	
	Steals	**0.53**					**0.57**				
Emotion	Somatic		**0.48**					**0.52**			
	Worries		**0.80**					**0.70**			
	Unhappy	**0.41**	**0.50**		0.34		0.31	**0.53**			
	Clingy		**0.58**					**0.63**			
	Afraid		**0.76**					**0.68**			
Hyperactivity	Restless			**0.74**					**0.67**		
	Fidgety			**0.74**					**0.65**		
	Distractible			**0.78**					**0.65**		
	Reflective			**0.53**		-0.35			**0.53**		
	Attends			**0.57**					**0.60**		
Peer Problems	Bullied				**0.49**					0.37	
	Popular				**0.70**					**0.56**	
	Good Friend				**0.71**					**0.56**	-0.36
	Loner				**0.56**		0.34			**0.60**	
	Relationships				**0.69**					**0.54**	
Prosocial	Considerate					**0.66**	-0.33				**0.61**
	Shares					**0.51**					**0.49**
	Caring					**0.76**					**0.70**
	Kind					**0.60**					**0.52**
	Helps out					**0.68**					**0.59**

#### Internal consistency of SDQ longitudinally

The internal consistency of the SDQ scales was assessed using Cronbach’s Alpha and McDonald’s Omega, the results were very similar using both statistics suggesting no bias in the Cronbach’s Alpha in this data (Table 3). For the total score this was good and was consistent across all time-periods, and in terms of both the parent and child questionnaires at 11 years. Within the sub-scales, the internal consistency was best for the hyperactivity sub-scale at all time-points, for the parent questionnaire and for the children’s self-report questionnaire at 11. In general, the least valid scale was the peer problems scale with an alpha of 0.53 at 3½ years of age, though this improved as the children got older. Notably it was also low from the children’s questionnaire at 11. Apart from the conduct sub-scale, the alphas were all larger at 7 and 11 than they were for the 3½ year old data. They were consistently lower in the children’s self-report data than the parental proxy reported data.

**Table 3:** Cronbach’s Alpha and McDonalds Omega for total difficulties score and sub-scales over time.

	Parent 3½		Parent 7		Parent 11		Child 11	
Scale	Alpha	Omega	Alpha	Omega	Alpha	Omega	Alpha	Omega
Total	0.78	0.76	0.79	0.77	0.82	0.82	0.77	0.77
Conduct	0.67	0.68	0.60	0.61	0.60	0.62	0.58	0.61
Emotion	0.58	0.59	0.67	0.68	0.67	0.69	0.64	0.65
Hyperactivity	0.72	0.75	0.80	0.81	0.77	0.77	0.68	0.69
Peer	0.53	0.54	0.59	0.58	0.67	0.67	0.49	0.50
Pro-social	0.64	0.64	0.70	0.71	0.70	0.71	0.58	0.59

#### Longitudinal associations of abnormal/borderline scores

This longitudinal study also allows an opportunity to assess the changes over time. Table 4, in the upper diagonal, shows that the correlation of the total difficulties scores between time points was moderate, and when the parent and child scores at 11 years. Similarly, the lower diagonal showing the agreement between time points for those with abnormal/borderline scores and shows low agreement in diagnosis between time points.

**Table 4:** Pearson correlations of total difficulty score over time (above diagonal) and kappa for abnormal/borderline vs normal (below diagonal).

	Parent 3½	Parent 7	Parent 11	Child 11
Parent 3½		0.58	0.51	0.24
Parent 7	0.39		0.63	0.32
Parent 11	0.26	0.33		0.51
Child 11	0.20	0.16	0.32	

Table 5 shows the incidence of abnormal and borderline cases for the total difficulties scale and the sub-scales at each point in time (using the definitions from the British data these should be 10% and 10% respectively [1]). The most notable result is the extremely high proportion of preschool children identified with conduct disorder. This over diagnosis appears to have corrected itself in the 7- and 11-year-old data, but conduct problems appear to remain relatively high compared to the other subscales. In general, the proportion of children identified with conduct and hyperactivity problems decreases over time, those with pro-social issues decrease over time and peer problem and emotional issues are lowest in the 7-year-old age group.

**Table 5:** Proportion of abnormal and borderline subject for the total difficulties scale and sub-scales by age.

	Parent 3½		Parent 7		Parent 11		Child 11	
	Ab	Bo	Ab	Bo	Ab	Bo	Ab	Bo
Total	12.2	10.9	4.6	7.6	5.7	7.5	10.3	11.5
Conduct	32.6	19.0	10.5	13.2	8.4	10.2	16.1	17.6
Hyperactivity	11.5	8.3	9.8	4.9	7.7	3.9	8.7	10.0
Peer	15.4	15.8	7.3	8.3	10.6	5.5	12.1	16.9
Emotion	8.1	6.4	7.1	4.8	11.4	5.8	13.4	12.9
Pro-social	4.1	10.6	3.6	6.8	2.4	6.7	4.2	7.7

Ab = Abnormal, Bo = Borderline.

#### Proportion of total difficulties by gender

We assessed differences in the total difficulties score by sex. The pattern remained the same at all ages with slightly more males than females having higher scores, though differences were only statistically significant at 7 years of age (3.5, 24.7% vs 21.7%, p = 0.35; 7 years, 15.0% vs 9.2%, p = 0.03; 11 years parent 15.5% vs 10.8%, p = 0.08; 11 years child 24.6% vs 19.0%. p = 0.09). Of note at 11 years is the lower than expected proportions reported from the parental perspective not to have problems, compared to the higher self-reported problems at this age, which are more in line with the expected norms.

## Discussion

Using the SDQ longitudinally across the same group of children has provided the opportunity to assess the validity, internal consistency, and reliability of the SDQ at various points in time but with the added advantage of using the same population of children. In general, we found the factor structure to be consistent over time, supporting earlier studies [11]. Similarly, the reliability of the Total Difficulties Score and each of the sub-scales is good and again in line with previously published cross sectional data [8, 9, 26]. There are several points of note, however, in relation to these analyses.

First, the question relating to obedience (“Generally obedient, usually does what adults request”) did not load highly at any of the time points in this sample of New Zealand children, only reaching a loading of 0.40 for the parental SDQ at 11. This was notably the lowest loading in the large cross sectional study of 10,000 English children by Goodman [7] and an Australian sample [8]. Thus, this question may be of limited use in identifying oppositional behaviour.

Secondly, we found a very high proportion of children scoring above the 90^th^ percentile for conduct problems at 3½ years of age. Although this high proportion was not found at ages 7 and 11 years, the proportion identified above the 90^th^ percentile for conduct problems at age 7 is higher than the other sub-scales and this is also the case for the child’s self-reported conduct problems at age 11 years. In contrast, the pro-social sub-scale had less than 5% scoring above the 90^th^ percentile across all time points.

There are two forms of the SDQ: one for use with children between the ages of 3–4 years and one for children older than four years of age. For both, the response key lies on a three-point Likert scale: Not at All True, Somewhat True, Certainly True. The two versions differ slightly in the conduct subscale. The questionnaire for younger children has items “often argumentative with adults” and “can be spiteful to others” which are replaced in the questionnaire for older children with the questions “often lies or cheats” and “steals from home, school or elsewhere”. In the present study, there are notable differences in the frequency scores for the corresponding questions at ages 3 and 7 years. For the “Often argumentative with adults” question, the relative frequencies for Somewhat True and Certainly True at age 3½ years are 47.3% and 11.4% and, for the comparative question at 7 years, they are 22.3% and 1.9%. For the “Can be spiteful to others” question, the relative frequencies of Somewhat True and Certainly True at age 3½ years are 26.3% and 2.8% compared to 2.9% and 1.0% at age 7 years for the comparative question. There are also relatively high frequencies for the “Often has temper tantrums or hot tempers” at age 3½ years (Somewhat True is 47.8% and Certainly True is 16.0%) compared to 36.7% and 10.5% at 7 years of age. The relatively high frequencies of these negative behaviours, particularly the “spiteful” comment, is surprising and are likely due to parental attribution about the intent of behaviour in preschool children. It is also possible that parents are interpreting these questions in a different way to which they were originally intended and tested.

The factor loadings for data collected at ages 7 and 11 years are generally higher than they are for the 3½-year-old data. There are notably more superfluous factors in the 3½-year-old factor analysis, suggesting that the SDQ is more reliable at these older ages.

Finally, it is of note that whilst the factor analyses remain consistent over time in this group of children, the children identified as having problems at the various time points changes as indicated by the relatively low correlations and kappa values. This is important as it shows the stability of the scale whislt the group of children identified as abnormal/borderline changes over time.

This study has some important limitations that need to be considered. First, as is common with cohort studies, and with many research studies more generally, the sample of children in this cohort is not representative of the whole population of children. However, as behavioural problems such as ADHD are considered to have a genetic basis, and given that the distribution of genes under the assumption of Mendelian randomisation, there is no reason to believe that the results of these analyses are not valid for all children in New Zealand.

Second, although the SDQ total difficulties score can be used as a continuous variable, it is common to categorise the scores into clinically meaningful groupings. For statistical reasons, we used the three-band categorisation to divide the data into those who are Normal versus children in the Borderline or Abnormal group. We acknowledge that there is now a fourth band to consider (splitting the apart the top 10% into two groups of high and very high scores; [27]), our sample size was not large enough to assess the validity of these new groups.

Across the scales that contribute to the total score, 11 year children self-report more difficulties than parents. This is understandable in that it could be argued parents are more likely to report difficulties in observable behaviour whilst self-report of children is also likely to include a reflection of internalising problems. The fact that children report increased difficulties across all and not just one domain e.g., emotional problems would indicate that they are not necessarily able to discriminate between internalising and externalising problems.

Finally, it could be argued that we should have used confirmatory factor analysis (CFA) rather than exploratory factor analysis (EFA). We determined that EFA was the more appropriate option for the purposes of this analysis for several reasons. Firstly, the SDQ was developed in the UK and has been validated in numerous other languages and countries; however, the questionnaire has not been shown to be valid across ages in New Zealand. Kersten et al have suggested that the factor structure of the SDQ is not valid in New Zealand at four to five years of age [28, 29], according to data collected as part of the New Zealand Before School Check. Unfortunately, there are some doubts about the quality of the data used in these analyses (personal communication), so the validity remains unclear.

We have previously also reported that a large proportion of children are defined as being abnormal on the conduct scale at pre-school age [24]. As such, our study is of particular importance in the New Zealand context. As the SDQ continues to be used by the Ministry of Health in New Zealand as part of the Before School Check. The results of our study are thus important for the interpretation of data from this child mental health and behavioural screening tool.

Secondly, because we have used the questionnaire longitudinally at three time points a changing factor structure over time would have an impact on how the SDQ was scored at different ages making longitudinal tracking difficult. Whilst there has been a suggestion of a modified five factor structure, the weightings of variables on factors and the diagnostics associated with CFA change very little between these two structures, and the modified structure removes the orthogonal nature of factors and calls for a different scoring system, which could vary by age making longitudinal tracking difficult. Additionally, as a number of papers have been published from this longitudinal dataset using the SDQ data at various ages, we feel that a potential change to the factor structure could produce confusing inconsistencies between manuscripts.

In conclusion, we found support for a five-factor SDQ model: Prosocial behaviour, emotional problems, conduct problems, peer problems, hyperactivity/inattention, that was stronger as our child participants grew older. The five-factor model was also consistent across all three time-periods and between the parent and child report at age 11 years. The moderate correlation between parent and child reports at age 11 (r = 0.51) is important information for both clinicians and researchers as is our finding that the proportion of children with high conduct and hyperactivity scores decreases over time. Finally, our finding that there is low agreement over the time from 3½ to 11 years of age in a child’s placement in the problematic/abnormal category of the SDQ Total Difficulties scores, should be reassuring to parents: behavioural problems can and do change over time.
